# Identification of key cuproptosis-related genes and their targets in patients with IgAN

**DOI:** 10.1186/s12882-022-02991-5

**Published:** 2022-11-03

**Authors:** Huagang Lin, Deping Wu, Jing Xiao

**Affiliations:** 1 Department of Nephrology, Huadong Hospital Affiliated to Fudan University, 200040 Shanghai, P.R. China; 2 Shanghai Key Laboratory of Clinical Geriatric Medicine, Huadong Hospital, Fudan University, Shanghai, P.R. China

**Keywords:** IgA nephropathy, Cuproptosis, Immune activity, Bioinformatics analysis

## Abstract

**Background:**

IgA nephropathy (IgAN) is one of the most common forms of chronic glomerulonephritis, but the aetiology and pathogenesis remain unclear. Cuproptosis is a newly identified form of cell death that plays an important role in many diseases. Researchers have not clearly determined whether the expression of cuproptosis-related genes (CRGs) is involved in the pathogenesis of IgAN.

**Methods:**

The GSE93798, GSE50469 and GSE37460 datasets containing microarray data from patients with IgAN (63) and healthy controls (31) were downloaded from the GEO database. Immune cells and immune-related functions were analysed in patients with IgAN and controls, and genes were identified that may be related to cuproptosis. A logistic regression model was established according to the results, and then GO and KEGG enrichment analyses were performed. Finally, possible drugs were selected using the DSigDB database.

**Results:**

The subjects in the different groups showed significantly different fractions of immune cells and immune-related functions, and 11 genes related to cuproptosis may be involved in these processes. Based on these 11 genes, the ROC curve was plotted, and the AUC value was calculated (0.898, 95% CI: 0.839–0.958). The result revealed good predictability. Then, genes with *P* < 0.05 (lipoyltransferase 1, LIPT1) were selected to plot an ROC curve, and the AUC value was calculated (0.729, 95% CI: 0.636–0.821). Enrichment analyses showed that the TCA cycle and multiple metabolic pathways may also be involved in the occurrence of IgAN. Finally, 293 potential drugs that may be used to treat IgAN were identified based on these genes.

**Conclusion:**

In this study, we identified some novel CRGs that may be involved in IgAN, among which LIPT1 was significantly differentially expressed. It may predict the risk of IgAN and provides a possible target for the treatment of IgAN. Further experimental studies are needed to explore how these CRGs mediate the occurrence and development of IgAN.

## Introduction

Immunoglobulin A nephropathy (IgAN) was initially described in 1968 by a French pathologist, Dr. Jean Berger, and his colleague, Dr. Nicole Hinglais (an electron microscopist), as a kidney disease with glomerular “intercapillary deposits of IgA-IgG” [1]. IgAN is characterized by dominant IgA glomerular deposits in renal biopsy that are usually accompanied by local cellular proliferation and matrix expansion [2]. It is also one of most common types of primary glomerulonephritis worldwide and remains a leading cause of chronic kidney disease and renal failure [3, 4]. An epidemiological survey of the global prevalence of IgAN in 2018 showed that the incidence of IgAN in Asian countries was significantly higher than that in Europe, the Americas and Africa. Among Asian countries, and the incidence rates in China and Japan were significantly higher than those in South Korea, India and other countries [5]. The clinical markers of the severity of kidney disease, including proteinuria, hypertension and impaired renal function, are nonspecific and only present when severe kidney injury occurs. Therefore, studies exploring the new possible pathogenic mechanisms of IgAN and identifying potential therapeutic targets are very important.

Copper (Cu) is an essential element required for the maintenance of the precise activities of eukaryotes, similar to iron (Fe) and zinc (Zn). Cu plays an important role in our body; on the one hand, Cu is a vital component of many mitochondrial enzymes, such as cytochrome c oxidase (COX) and superoxide dismutase (SOD1) [6, 7]. On the other hand, mitochondria are central for Cu metabolism [8]. The term “cuproptosis” was proposed by Tsvetkov et al [9]. They defined it as a Cu-elesclomol-triggered, ferredoxin-dependent form of cell death distinct from other modalities of cell death, such as apoptosis, ferroptosis, necroptosis, and pyroptosis [9, 10]. Recently, cuproptosis has been recognized as a possibly new mechanism in some diseases [9]. However, researchers have not determined whether cuproptosis is involved in the development of IgAN.

At present no studies have examined whether cuproptosis is involved in the pathogenesis of IgAN. In this study, we screened the pyroptosis-related genes (CRGs) that may be involved in IgAN according to the data in three GEO databases, established models and screened some possible therapeutic drugs.

## Materials and methods

### Acquisition and preprocessing of public data

Three microarray datasets of IgAN (GSE93798, GSE50469 and GSE37460) were downloaded from the GEO database (http://www.ncbi.nlm.nih.gov/geo/) using “IgAN” as the search term. Ninety-four samples were obtained from glomerular tissue, including 63 from patients with IgAN and 31 from healthy people. Since cuproptosis is a new concept and no relevant database is available, cuproptosis-related genes were selected according to the article by Peter Tsvetkov et al. [9]. Thirteen CRGs were identified. The immune matrix was downloaded from GESA (http://www.gsea-msigdb.org/) and divided into immune cell- and immune-related functions.

### Data processing and construction of the heatmap

The “limma”, “ggpubr” and “reshape2” R packages were used to combine IgAN, cuproptosis and immune matrix. The “Pheatmap” R package was used to plot an immune heatmap. The “corrplot” R package was used to analyse and plot the correlation of the immune matrix.

### Differences in immune activity between the IgAN and control groups

The “ggpubr” and “reshape2” R packages were used to determine the differences in immune cells and functions between the IgAN and control groups, and *P* < 0.05 was considered statistically significant.

### Selection of CRGs associated with IgAN

The “psych” and “ggcorrplot” R packages were installed to analyse the correlation between the cuproptosis-related gene matrix and IgAN immune matrix, and a heatmap was drawn.

### Model building and analysis

According to the mean value of gene expression, the data in the matrix were divided into low expression and high expression. The “rms” R package was used to build a logit model and establish a nomogram. The c-index was calculated, and the ROC curve was plotted using the “ROCR” R package. The nonadherence nomogram was subjected to bootstrapping validation (50 bootstrap resamples) to calculate a relatively corrected *C*-index.

### Functional enrichment analyses and screening of possible drugs

Gene Ontology (GO) and Kyoto Encyclopedia of Genes and Genomes (KEGG) analyses were performed using R software. Possible drugs for the treatment of IgAN were analysed using the DSigDB database (https://maayanlab.cloud/Enrichr/).

### Statistical analysis

All statistical analyses and preparation of figures were implemented using R 4.2.0 (R Foundation, Vienna, Austria). A 95% confidence interval (CI) was evaluated by performing univariable and multivariate logistic regression analyses. If not specified above, *p* < 0.05 was considered statistically significant.

## Results

### The immune activity of patients with IgAN has changed significantly

**Fig. 1:**
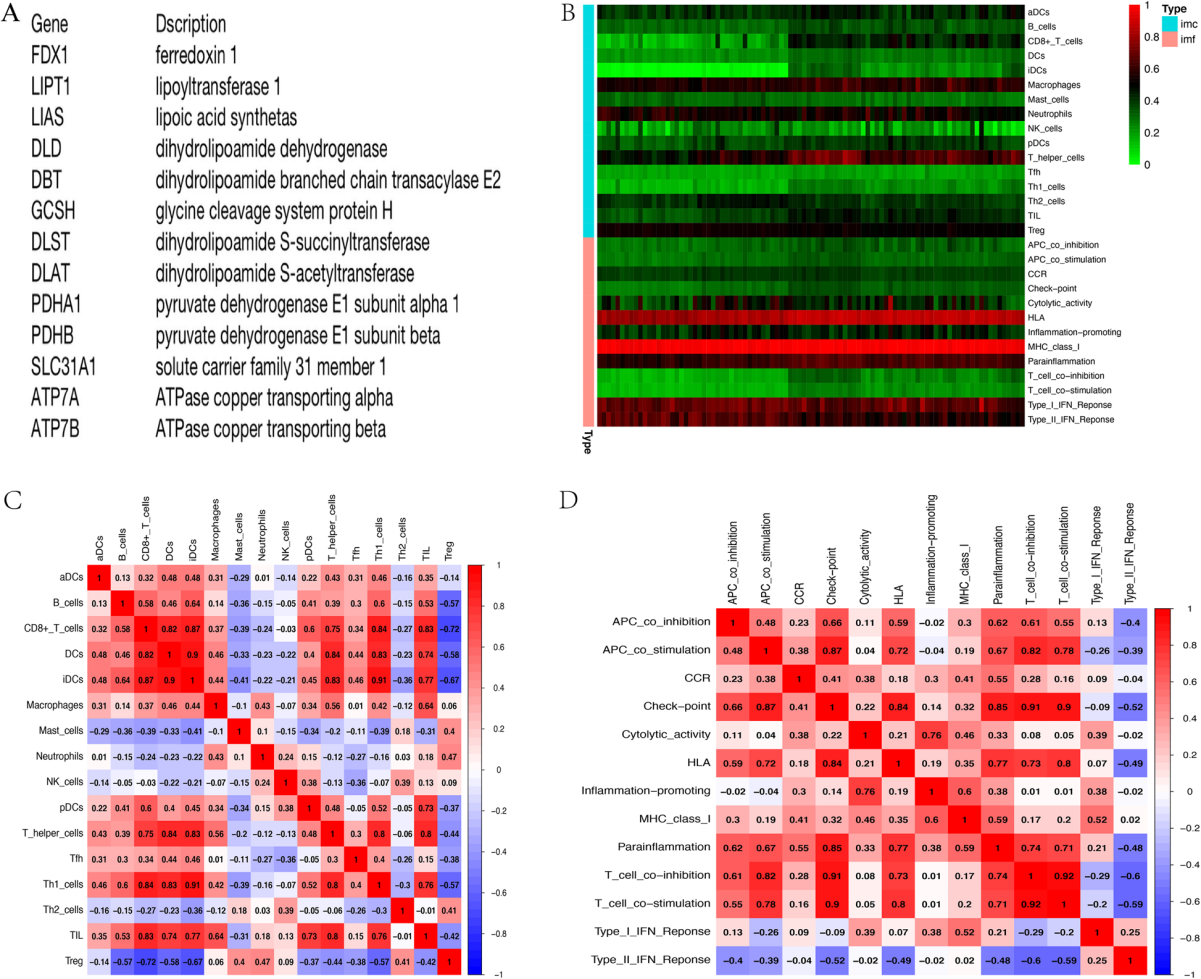
CRGs and immune heatmap. **A** CRGs selected from references. **B** Heatmap based on immune activity in the GEO database and CRGs. **C** Heatmap of the correlation between immune cells. **D** Heatmap of the correlation between immune-related functions. CRGs: cuproptosis-related genes

As described above, we selected 13 CRGs for study (Fig. 1A). We analysed the data to explore the changes in immune cells and immune-related functions of CRGs in patients with IgAN. Significant changes in immune activity occurred in patients with IgAN, and many immune cells and immune-related functions were changed (Fig. 1B). In addition, we plotted correlation heatmaps to understand the correlations between immune cells and immune-related functions (Fig. 1C, D). The results showed correlations between them, and the numbers in the heatmap represent the correlation coefficient.

### Analysis of disease and immunity

**Fig. 2:**
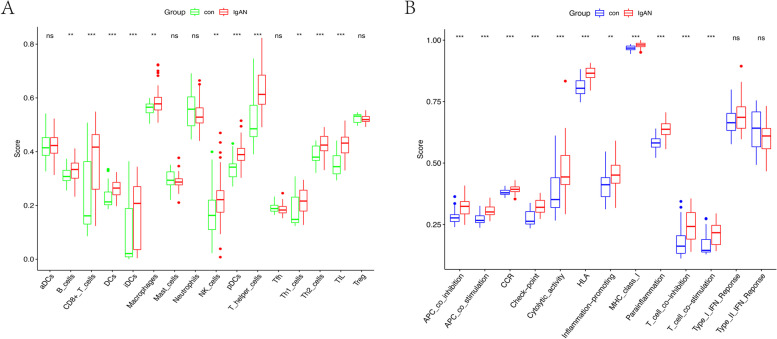
Analysis of disease and immune activity. **A** Comparison of the immune cell results between control and IgAN patients. **B** Comparison of the immune-related function results between the two groups. ***, *p* < 0.001; **, *p* < 0.01; *, *p* < 0.05; ns, *p* > 0.05

The analysis compared immune activity between the IgAN group and the control group using three GEO datasets based on scores for 16 types of immune cells and 13 types of immune-related functions. The results (Fig. 2 A, B) showed significant differences in immune activity between the two groups, especially in CD8 + T cells, dendritic cells (DCs), iDCs, pDCs, T helper cells, Th2 cells, tumour-infiltrating lymphocytes (TIL), antigen-presenting cell (APC) coinhibition, APC costimulation, CCR, checkpoint, cytolytic activity, human leukocyte antigen (HLA), major histocompatibility complex (MHC) class I, parainflammation, T cell coinhibition and T cell costimulation, with *P* values < 0.001. Immune activity essentially differed between the two groups.

### Correlation between CRGs and immunity activity

**Fig. 3:**
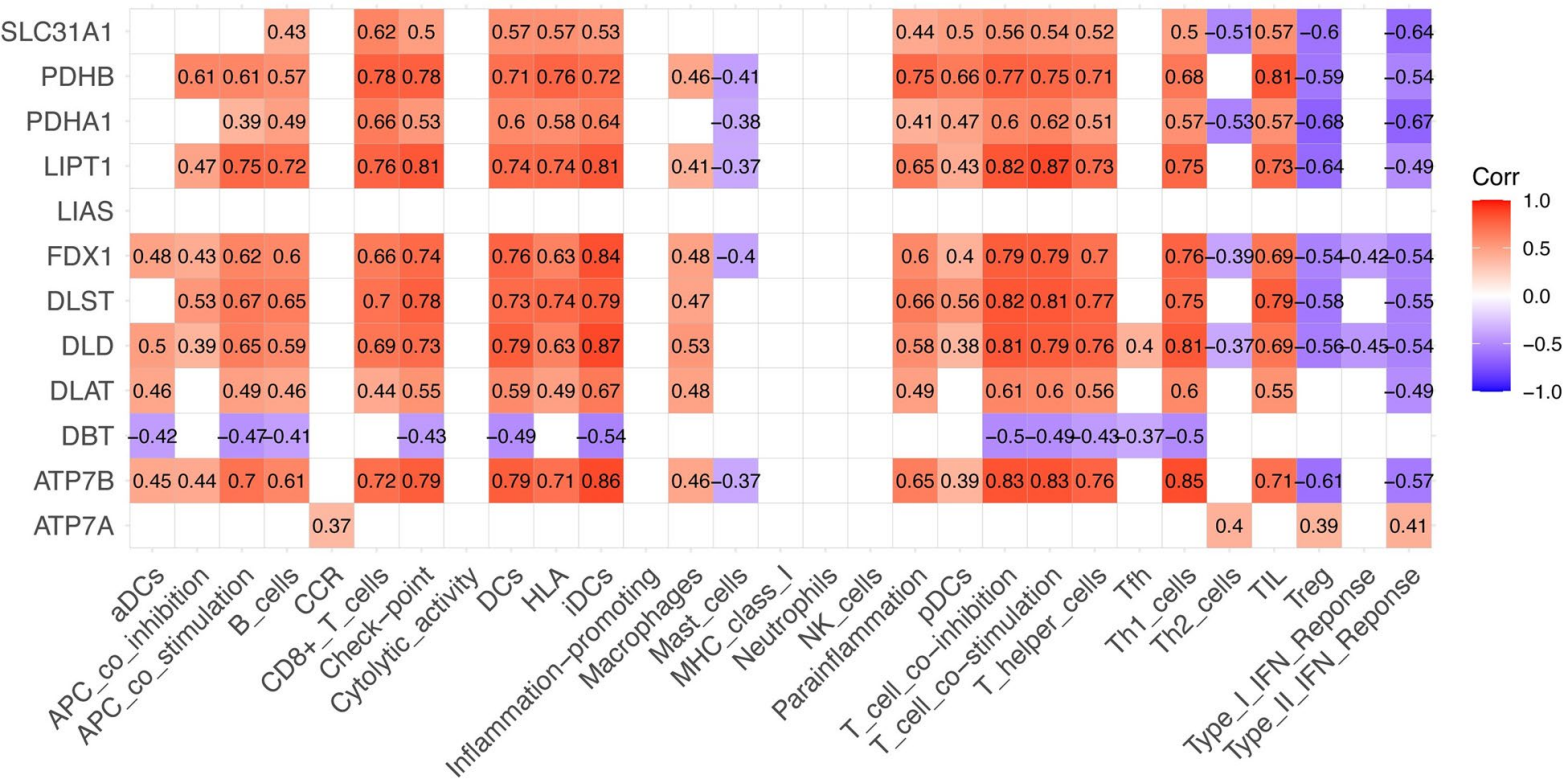
Correlation analysis between CRGs and immune activity. Only statistically significant correlation coefficients are shown

We analysed the correlation between the CRG matrix and immune matrix and plotted a heatmap to select CRGs that may be involved in IgAN. As shown in Fig. 3, significant correlations were observed between solute carrier family 31 member 1 (SLC31A1), pyruvate dehydrogenase E1 subunit beta (PDHB), pyruvate dehydrogenase E1 subunit alpha 1 (PDHA1), lipoyltransferase 1 (LIPT1), ferredoxin 1 (FDX1), dihydrolipoamide S-succinyltransferase (DLST), dihydrolipoamide dehydrogenase (DLD), dihydrolipoamide S-acetyltransferase (DLAT), dihydrolipoamide branched chain transacylase E2 (DBT), ATPase copper transporting beta (ATP7B) and ATPase copper transporting alpha (ATP7A).

### Construction of models

**Fig. 4:**
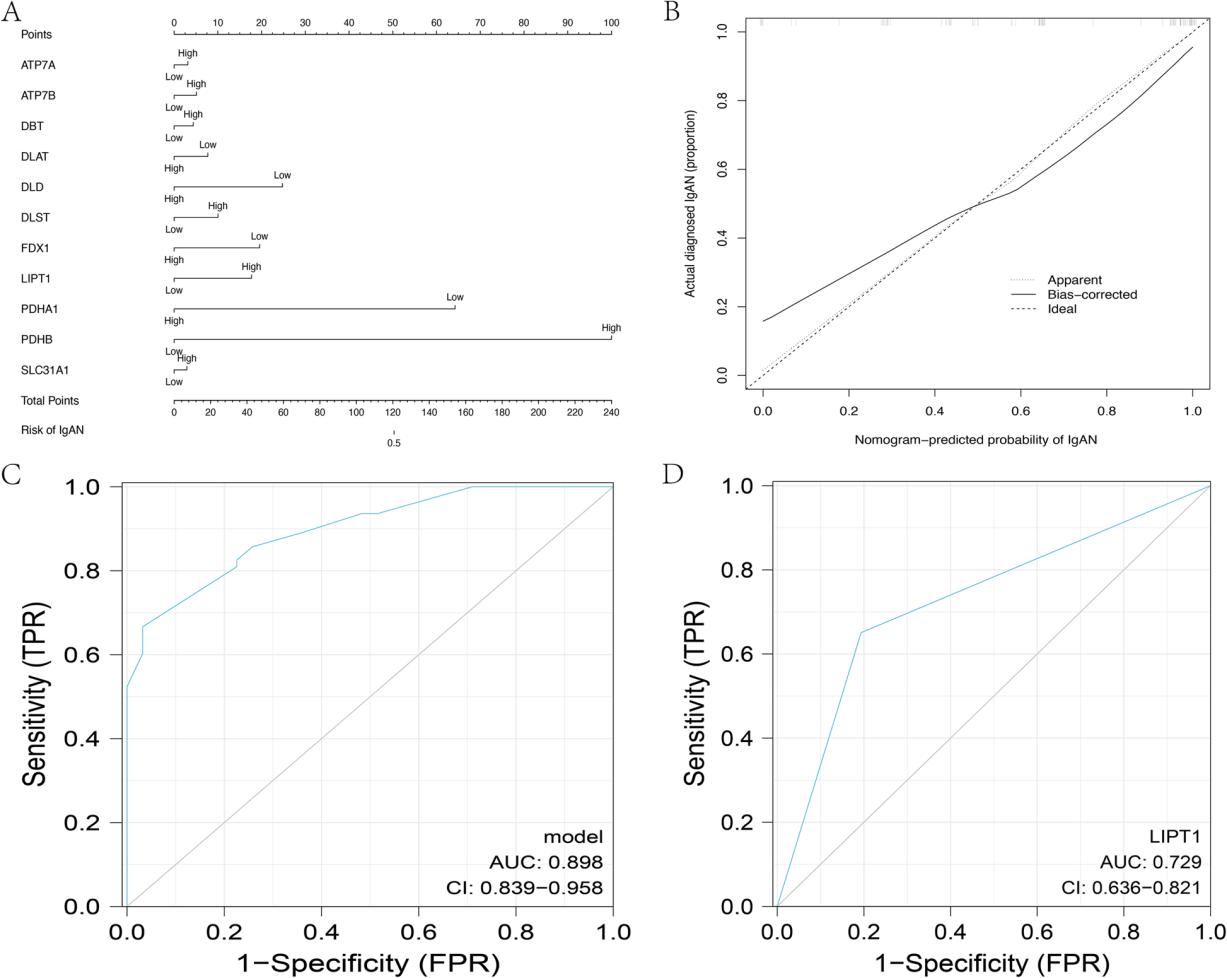
Nomogram and logistic models. **A** A nomogram to predict the risk of occurrence of IgAN. **B** The calibration curve of the logistic regression model. **C** Total ROC curve of the model. **D** The LIPT1 ROC curve

We divided the two groups of data into high expression and low expression according to the median values. After the data were processed, the 11 genes shown in Fig. 3 were used for the logistic regression analysis, obtaining 3 pyroptosis-related genes with *p* < 0.3, and significant differences in LIPT1 expression were observed (Table 1). The nomogram and calibration curve were plotted. The total C-index was 0.898 (95% CI: 0.839–0.958), and the validated C-index was 0.840 (Fig. 4A, B). A total ROC curve for these genes was plotted, and the AUC value was calculated, which showed that the model had a good prediction effect (Fig. 4C). Since the calculated OR for each of these seven genes was greater than 1, they were judged as being high-risk genes that may be positively associated with the occurrence of IgAN, while the other four genes were negatively associated with the occurrence of IgAN, and one of them (LIPT1) was identified as an independent gene associated with IgAN occurrence with *p* < 0.05. Therefore, LIPT1 was selected to plot a ROC curve (Fig. 4D).

**Table 1:** Logistic regression analysis of CRGs in the three GEO databases and the C-index

Gene	Median	Coefficient	OR	AUC	95% CI	*P* value
ATP7A	6.705	0.313	1.871	0.536	0.428–0.645	0.388
ATP7B	6.996	0.511	2.777	0.680	0.582–0.779	0.616
DBT	5.758	0.438	2.400	0.512	0.403–0.621	0.213
DLAT	7.112	-0.773	0.213	0.560	0.452–0.668	0.108
DLD	8.911	-7.039	7.690e-7	0.680	0.582–0.779	0.996
DLST	7.689	1.006	7.474	0.753	0.665–0.841	0.490
FDX1	8.511	-6.517	2.184e-6	0.705	0.609-0.800	0.997
LIPT1	7.824	1.733	34.667	0.729	0.636–0.821	0.021*
PDHA1	9.216	-10.999	2.798e-10	0.632	0.529–0.736	0.992
PDHB	10.451	23.697	3.832e + 20	0.777	0.693–0.860	0.992
SLC31A1	7.4360	0.293	1.796	0.608	0.503–0.714	0.629

CRG logistic regression analysis.

*CRGs* Cuproptosis-related genes, *OR* Odds ratio, 95% CI: 95% confidence interval

*P < 0.05

### GO and KEGG Enrichment analyses of overlapping genes

**Fig. 5:**
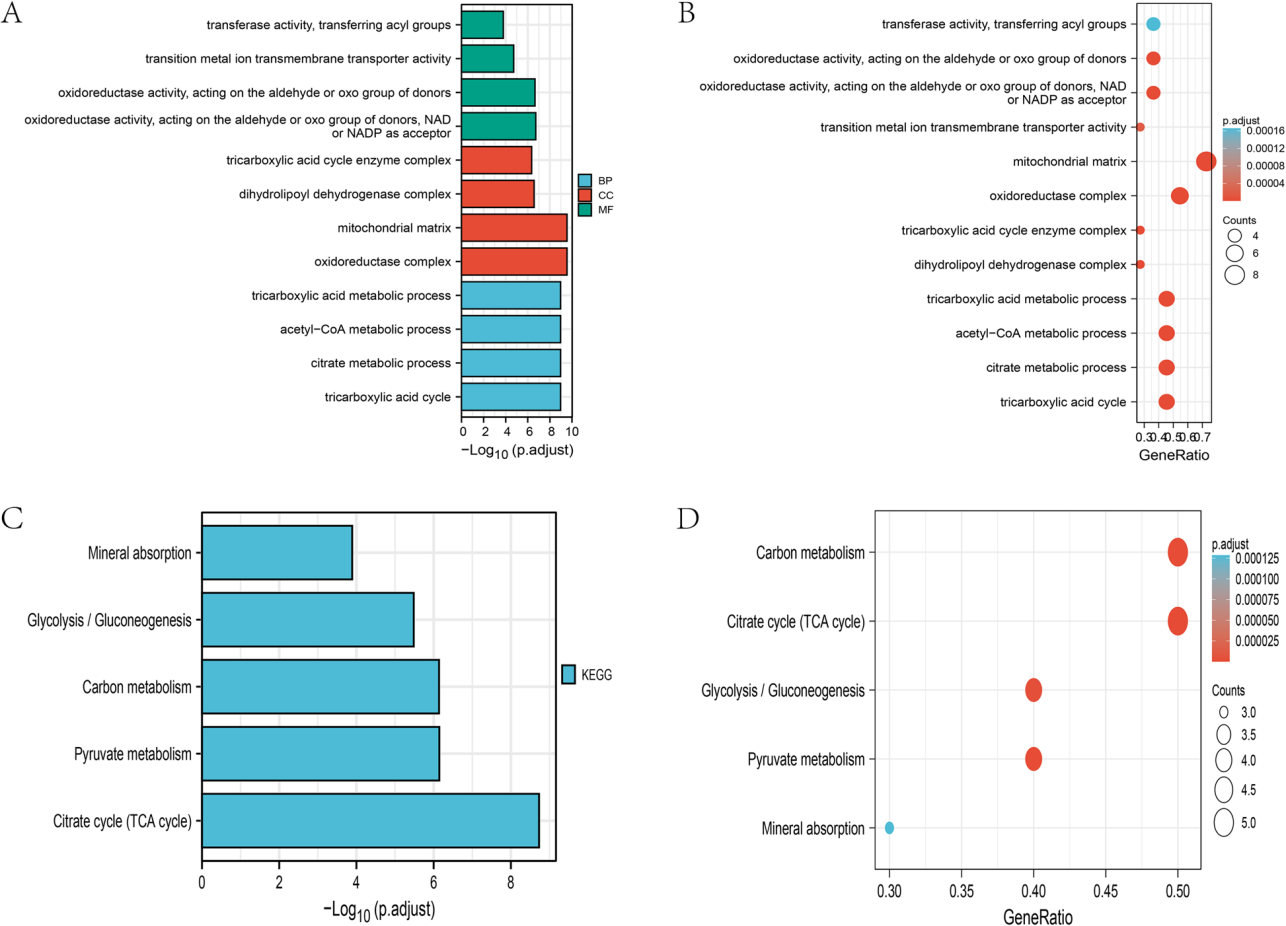
GO and KEGG enrichment analyses of CRGs. **A** Bar charts of the GO functional analysis of CRGs. **B** Bubble diagrams of the GO functional analysis of CRGs. **C** Bar charts of the KEGG pathway enrichment analysis of CRGs. **D** Bubble diagrams of the KEGG pathway enrichment analysis of CRGs.

We selected 11 CRGs that may be involved in the occurrence of IgAN for GO (Fig. 5A, B) and KEGG enrichment analyses (Fig. 5C, D). The enrichment results for these genes showed that they were involved in many metabolic pathways, including glycolysis, the TCA cycle, and carbohydrate metabolism, and mitochondria were highly enriched in these genes.

### Targeted drug prediction

We used the DSigDB database to predict potential target drugs associated with key genes that may be useful for treating IgAN by regulating cuproptosis. A total of 293 possible drugs were predicted. The top 10 predicted target drugs according to the combined scores are shown in Table 2.

**Table 2:** Top 10 predicted target drugs

Index	Name	*P* value	Odds Ratio	Combined Score
1	Vitinoin CTD 00007069	1.35e-06	29.79069767	402.6719766
2	Bathocuproine disulfonate CTD 00001350	9.94e-06	96.97402597	1117.079896
3	DIAMIDE CTD 00005785	4.68e-05	261.0718954	2602.868539
4	1,6,7,8,9,11a,12,13,14,14a-Decahydro-1,13-dihydroxy-6-methyl-4 H-cyclopent [f]oxacyclotridecin-4-one CTD 00007351	2.02e-04	119.8318318	1019.71877
5	D-Penicillamine CTD 00006475	3.19e-04	94.28841608	759.0882618
6	Chlorzoxazone HL60 DOWN	3.63e-04	12.17017955	96.39489077
7	Latamoxef HL60 DOWN	9.37e-04	9.756304302	68.02977945
8	Adenosine triphosphate CTD 00005324	9.58e-04	53.29585007	370.4623059
9	Valproic acid CTD 00006977	0.00104922	14.07733076	96.56641374
10	Atrazine CTD 00005450	0.00250277	6.898176907	41.32253183

## Discussion

In this study, we screened 11 CRGs that may be involved in the occurrence and development of IgAN by analysing three GEO databases. Then, we constructed a logistic regression model for these genes and drew a nomogram and a ROC curve; the results indicated that these genes may be involved in the occurrence of IgAN. We selected the gene with a significant difference in expression (LIPT1) to draw the ROC curve, with an AUC area of 0.729, suggesting that it is a good predictor of the risk of IgAN. Some studies have found that LIPT1 may lead to the activation of the NOD-like receptor thermal protein domain associated protein 3 (NLRP3) pathway by affecting mitochondrial function and the TCA cycle, activating the immune response, and promoting the occurrence of IgAN [11, 12]. In addition, we performed enrichment analyses of these genes, and the results showed that these genes were mainly involved in a variety of metabolic pathways in vivo. Finally, using these genes, we screened relevant drugs that may be used to treat IgAN in the future. Compared with previous studies on patients with IgAN, we first analysed the differences in CRG expression in patients with IgAN and healthy people, established a model and screened potential therapeutic drugs. We screened 293 possible drugs. Interestingly, D-penicillamine was used to treat a patient with IgA nephropathy with Wilson’s disease. After three months of treatment, the patient’s urinary protein level was significantly improved [13].

The main causes of IgAN may be the deposition of glomerular IgA immune complexes (ICS) or IgA immune aggregates and the activation of innate immunity, followed by the activation of T cells and the resulting inflammatory response [14, 15]. However, the exact pathogenesis of IgAN remains unclear, and the specific treatment of renal disease is still insufficient. The internationally recognized theoretical basis of IgAN is the “four hit” hypothesis [1]: ① the serum level of galactose-deficient IgA1 (GD-IgA1) in the circulation is significantly increased; ② GD-IgA1 is recognized by an IgG antibody in vivo to induce the production of autoantibodies; ③ antigen–antibody complexes are formed and widely deposited in mesangial cells; and ④ mesangial cell proliferation, glomerular inflammation and injury occur [16]. These immune complexes may be deposit in the glomerular mesangial area and then activate mesangial cells, which damage kidney tubular cells and podocytes (apoptosis and foot effacement) by secreting many detrimental bioactive mediators, such as IL-6, tumour necrosis factor (TNF), aldosterone, transforming growth factor-β (TGFβ), and angiotensin II (Ang II) [17]. However, this hypothesis also has some limitations. For example, it does not explain why GD-IgA1 also exists in the circulation of healthy individuals, and IgA deposition has also been observed in the glomerular mesangium [18]. Therefore, a better understanding of the pathogenesis of IgAN may help to identify new targets and better treat patients with IgAN.

The aetiology of IgAN consists of many factors, among which immunity plays an important role in the pathogenesis of IgAN. T cell subsets are commonly measured in the clinic to reflect the state of immune function. The immunoregulatory function of T cells is mainly completed by CD4 + T cells and CD8 + T cells. Some studies have documented an increase in the number of CD8 + T cells in patients with IgAN [19], consistent with our results. The cytotoxic effect of CD8 + T cells may accelerate damage to the glomerulus. The synthesis and secretion of low glycosylated IgA1 are regulated by Th cells. Many studies have found that an imbalance in the Th1/Th2 cell ratio leads to an increase in the number of Th2 cells, which might lead to the synthesis of low glycosylated IgA1 and a sustained immune response in a variety of pathways [20, 21]. In our study, we also observed significant differences in the numbers of Th1 cells and Th2 cells in glomerular samples between patients with IgAN and healthy people. However, we did not analyse the changes in Th1/Th2. Our study also found that B cells may also be involved in the occurrence of IgAN. Some studies suggest that B cells may promote the secretion of GD-IgA1 through Toll-like receptors (TLRs) and a proliferation-inducing ligand (APRIL) [22]. Consistent with our findings, some studies have found that natural killer (NK) cells and dendritic cells (DCs) may participate in the development of IgAN [19], and more mechanisms require further exploration. In adults and children with IgAN, a larger number of CD68 + cells (markers of macrophages) were correlated with worse outcomes [23, 24]. We also observed a significant increase in the number of macrophages in patients with IgAN compared with healthy people. However, we did not detect a difference in the number of glomerular Treg cells between patients with IgAN and healthy people, suggesting that Treg cells may not play a significant role in IgAN glomerulopathy. Our results also show that a variety of immune-related functions are altered in patients with IgAN. In the present study, we found that LIPT1 may be involved in IgAN. Recently, LIPT1 was reported to affect fatty acid synthesis by regulating metabolism. For example, increased expression of LIPT1 may promote fatty acid synthesis and reduce fatty acid oxidation [12]. In addition, some researchers have shown that P-aIgA1 (aggregated IgA1 from patients with IgAN) promotes the proliferation of human kidney mesangial cells, while the administration of short-chain fatty acid inhibitors reverses the increased proliferation of mesangial cells and extracellular matrix synthesis [25]. However, few relevant studies are available, and further evidence is needed to determine how LIPT1 participates in IgAN.

In recent years, progress has been achieved in determining how essential trace metals exert toxic effects on mammalian cells. These different metals kill cells by activating a process that does not require an apoptotic pathway. For example, excess zinc triggers nonapoptotic cell death by inhibiting the synthesis of adenosine triphosphate (ATP) [26, 27]. Iron catalyses the formation of toxic membrane lipid peroxides. Iron sagging is a unique form of nonapoptotic cell death [28]. Copper and other trace metals are essential for life, but research has revealed that copper also causes cell death [29]. Copper-binding agents have been used to treat cancer because they bind copper and cause cancer cell death. A drug forming a bond with copper called elesclomol has been used in clinical trials to treat cancer, and although the success rate is limited [30, 31], it provides a new idea for the treatment of cancer in the future. However, the mechanism of cuproptosis is not yet clear. Evidence from yeast and mammalian cells suggests that copper toxicity is related to changes in mitochondrial function [10]. Our GO enrichment analysis also showed that these CRGs were enriched in the mitochondrial matrix, suggesting that mitochondria play an important role in cuproptosis. According to a previous study, cuproptosis is activated by mitochondrial stress, which is characterized by the assembly of lipoylated mitochondrial enzymes and the reduction of Fe-S cluster proteins rather than mitochondrial reactive oxygen species (ROS) [32]. A recent study found that copper directly binds to fatty acylated components in the tricarboxylic acid (TCA) cycle, leading to the accumulation of fatty acylated proteins and the subsequent loss of sulfur cluster proteins, leading to protein toxic stress and eventually cell death [9]. study indicates that the TCA cycle plays an important role in cuproptosis, consistent with the results of our enrichment analysis. Copper mediates a variety of kidney diseases [33–36], and the use of copper bonds can treat renal injury caused by cisplatin [37]. These studies show that copper may participate in the occurrence and development of some kidney diseases through a variety of pathways. Therefore, research on cuproptosis-mediated kidney diseases may become a new hotspot in future kidney disease research.

Our research has several advantages, as described below. First, we systematically analysed the expression of CRGs in patients with IgAN and constructed a model based on these databases to predict the risk of IgAN. Second, we suggested that a correlation may exist between cuproptosis and immune changes in the process of IgAN occurrence and development, which provides a new direction for the treatment and research of IgAN. However, the study also has some limitations. First, the sample size in the database is not sufficient, which may lead to errors. In addition, we did not conduct in vivo and in vitro experiments to verify these results, and further research is needed to reveal the potential mechanism of cuproptosis in IgAN.

## Conclusion

In conclusion, this study systematically analysed the immune changes related to cuproptosis in patients with IgAN. Our study shows that these CRGs may play a crucial role in the occurrence and development of IgAN. Our results provide a new direction for IgAN research and treatment in the future.

## Data Availability

Publicly available datasets were analyzed in this study. These data can be found here: https://www.ncbi.nlm.nih.gov/geo/, GSE93798, GSE50469 and GSE37460.

## Abbreviations

IgAN: Immunoglobulin A nephropathy
GWAS: genome-wide association study
COX: cytochrome c oxidase
SOD1: superoxide dismutase 1
CRGs: cuproptosis-related genes
GO: Gene Ontology
KEGG: Kyoto Encyclopedia of Genes and Genomes
ICS: IgA immune complexes
GD-IgA1: galactose-deficient IgA1
TNF: tumour necrosis factor
TGFβ: transforming growth factor-β
AngII: angiotensin II
TLRs: Toll-like receptors
NK: natural killer
DCs: dendritic cells
ATP: adenosine triphosphate
ROS: reactive oxygen species
TCA: tricarboxylic acid
LIPT1: lipoyltransferase 1
NLRP3: NOD-like receptor thermal protein domain associated protein 3

## References

[1] Berger J, Hinglais N (1968). [Intercapillary deposits of IgA-IgG]. J Urol Nephrol (Paris).

[2] Roberts IS. Pathology of IgA nephropathy. Nat Rev Nephrol. 2014;10(8):445–54. 10.1038/nrneph.2014.92 24861083

[3] Rovin BH, Adler SG, Barratt J, Bridoux F, Burdge KA, Chan TM (2021). Executive summary of the KDIGO 2021 Guideline for the Management of Glomerular Diseases. Kidney Int.

[4] Wyatt RJ, Julian BA (2013). IgA nephropathy. N Engl J Med.

[5] Schena FP, Nistor I (2018). Epidemiology of IgA Nephropathy: A Global Perspective. Semin Nephrol.

[6] Leary SC (2010). Redox regulation of SCO protein function: controlling copper at a mitochondrial crossroad. Antioxid Redox Signal.

[7] Ilyechova EY, Bonaldi E, Orlov IA, Skomorokhova EA, Puchkova LV, Broggini M. CRISP-R/Cas9 Mediated Deletion of Copper Transport Genes CTR1 and DMT1 in NSCLC Cell Line H1299. Biological and Pharmacological Consequences. Cells. 2019;8(4):322. 10.3390/cells8040322 PMC6523758 30959888

[8] Cobine PA, Moore SA, Leary SC (2021). Getting out what you put in: Copper in mitochondria and its impacts on human disease. Biochim Biophys Acta Mol Cell Res.

[9] Tsvetkov P, Coy S, Petrova B, Dreishpoon M, Verma A, Abdusamad M (2022). Copper induces cell death by targeting lipoylated TCA cycle proteins. Science.

[10] Tsvetkov P, Detappe A, Cai K, Keys HR, Brune Z, Ying W (2019). Mitochondrial metabolism promotes adaptation to proteotoxic stress. Nat Chem Biol.

[11] Tsai YL, Hua KF, Chen A, Wei CW, Chen WS, Wu CY (2017). NLRP3 inflammasome: Pathogenic role and potential therapeutic target for IgA nephropathy. Sci Rep.

[12] Ni M, Solmonson A, Pan C, Yang C, Li D, Notzon A (2019). Functional Assessment of Lipoyltransferase-1 Deficiency in Cells, Mice, and Humans. Cell Rep.

[13] Bhandari G, Tiwari V, Gupta A, Gupta P, Bhargava V, Malik M (2021). IgA Nephropathy with Wilson’s Disease: A Case Report and Literature Review. Indian J Nephrol.

[14] Coppo R, Amore A, Peruzzi L, Vergano L, Camilla R (2010). Innate immunity and IgA nephropathy. J Nephrol.

[15] Rifai A (2007). IgA nephropathy: immune mechanisms beyond IgA mesangial deposition. Kidney Int.

[16] Rajasekaran A, Julian BA, Rizk DV (2021). IgA Nephropathy: An Interesting Autoimmune Kidney Disease. Am J Med Sci.

[17] Pattrapornpisut P, Avila-Casado C, Reich HN (2021). IgA Nephropathy: Core Curriculum 2021. Am J Kidney Dis.

[18] Gale DP, Molyneux K, Wimbury D, Higgins P, Levine AP, Caplin B (2017). Galactosylation of IgA1 Is Associated with Common Variation in C1GALT1. J Am Soc Nephrol.

[19] Esteve Cols C, Graterol Torres FA, Quirant Sánchez B, et al. Immunological Pattern in IgA Nephropathy. Int J Mol Sci. 2020;21(4):1389. 10.3390/ijms21041389 PMC7073027 32085673

[20] Good KL, Avery DT, Tangye SG (2009). Resting human memory B cells are intrinsically programmed for enhanced survival and responsiveness to diverse stimuli compared to naive B cells. J Immunol.

[21] Stavnezer J, Kang J (2009). The surprising discovery that TGF beta specifically induces the IgA class switch. J Immunol.

[22] Chang S, Li XK (2020). The Role of Immune Modulation in Pathogenesis of IgA Nephropathy. Front Med (Lausanne).

[23] Ikezumi Y, Suzuki T, Imai N, Ueno M, Narita I, Kawachi H (2006). Histological differences in new-onset IgA nephropathy between children and adults. Nephrol Dial Transplant.

[24] Kawasaki Y, Suyama K, Miyazaki K, Kanno S, Ono A, Suzuki Y (2014). Resistance factors for the treatment of immunoglobulin A nephropathy with diffuse mesangial proliferation. Nephrol (Carlton).

[25] Dai Q, Liu J, Du YL, Hao X, Ying J, Tan Y (2016). Histone deacetylase inhibitors attenuate P-aIgA1-induced cell proliferation and extracellular matrix synthesis in human renal mesangial cells in vitro. Acta Pharmacol Sin.

[26] Dineley KE, Votyakova TV, Reynolds IJ (2003). Zinc inhibition of cellular energy production: implications for mitochondria and neurodegeneration. J Neurochem.

[27] Du W, Gu M, Hu M, Pinchi P, Chen W, Ryan M (2021). Lysosomal Zn(2+) release triggers rapid, mitochondria-mediated, non-apoptotic cell death in metastatic melanoma. Cell Rep.

[28] Kagan VE, Mao G, Qu F, Angeli JP, Doll S, Croix CS (2017). Oxidized arachidonic and adrenic PEs navigate cells to ferroptosis. Nat Chem Biol.

[29] Ge EJ, Bush AI, Casini A, Cobine PA, Cross JR, DeNicola GM (2022). Connecting copper and cancer: from transition metal signalling to metalloplasia. Nat Rev Cancer.

[30] O’Day SJ, Eggermont AM, Chiarion-Sileni V, Kefford R, Grob JJ, Mortier L (2013). Final results of phase III SYMMETRY study: randomized, double-blind trial of elesclomol plus paclitaxel versus paclitaxel alone as treatment for chemotherapy-naive patients with advanced melanoma. J Clin Oncol.

[31] Monk BJ, Kauderer JT, Moxley KM, Bonebrake AJ, Dewdney SB, Secord AA (2018). A phase II evaluation of elesclomol sodium and weekly paclitaxel in the treatment of recurrent or persistent platinum-resistant ovarian, fallopian tube or primary peritoneal cancer: An NRG oncology/gynecologic oncology group study. Gynecol Oncol.

[32] Tang D, Chen X, Kroemer G (2022). Cuproptosis: a copper-triggered modality of mitochondrial cell death. Cell Res.

[33] Liao J, Yang F, Bai Y, Yu W, Qiao N, Han Q (2021). Metabolomics analysis reveals the effects of copper on mitochondria-mediated apoptosis in kidney of broiler chicken (Gallus gallus). J Inorg Biochem.

[34] Liao J, Yang F, Yu W, Qiao N, Zhang H, Han Q (2020). Copper induces energy metabolic dysfunction and AMPK-mTOR pathway-mediated autophagy in kidney of broiler chickens. Ecotoxicol Environ Saf.

[35] Niu YY, Zhang YY, Zhu Z, Zhang XQ, Liu X, Zhu SY (2020). Elevated intracellular copper contributes a unique role to kidney fibrosis by lysyl oxidase mediated matrix crosslinking. Cell Death Dis.

[36] Wan F, Zhong G, Ning Z, Liao J, Yu W, Wang C (2020). Long-term exposure to copper induces autophagy and apoptosis through oxidative stress in rat kidneys. Ecotoxicol Environ Saf.

[37] Khairnar SI, Mahajan UB, Patil KR, Patel HM, Shinde SD, Goyal SN (2020). Disulfiram and Its Copper Chelate Attenuate Cisplatin-Induced Acute Nephrotoxicity in Rats Via Reduction of Oxidative Stress and Inflammation. Biol Trace Elem Res.

